# The Temporal Relationship Between Ecological Pain and Life-Space Mobility in Older Adults With Knee Osteoarthritis: A Smartwatch-Based Demonstration Study

**DOI:** 10.2196/19609

**Published:** 2021-01-13

**Authors:** Mamoun T Mardini, Subhash Nerella, Matin Kheirkhahan, Sanjay Ranka, Roger B Fillingim, Yujie Hu, Duane B Corbett, Erta Cenko, Eric Weber, Parisa Rashidi, Todd M Manini

**Affiliations:** 1 Department of Aging and Geriatric Research University of Florida Gainesville, FL United States; 2 Department of Health Outcomes and Biomedical Informatics University of Florida Gainesville, FL United States; 3 Department of Biomedical Engineering University of Florida Gainesville, FL United States; 4 Google LLC Mountain View, CA United States; 5 Department of Computer and Information Science and Engineering University of Florida Gainesville, FL United States; 6 Department of Community Dentistry and Behavioral Science University of Florida Gainesville, FL United States; 7 Department of Geography University of Florida Gainesville, FL United States; 8 Department of Epidemiology University of Florida Gainesville, FL United States

**Keywords:** ecological momentary assessment, smartwatch app, life-space mobility, pain, knee osteoarthritis, global positioning system

## Abstract

**Background:**

Older adults who experience pain are more likely to reduce their community and life-space mobility (ie, the usual range of places in an environment in which a person engages). However, there is significant day-to-day variability in pain experiences that offer unique insights into the consequences on life-space mobility, which are not well understood. This variability is complex and cannot be captured with traditional recall-based pain surveys. As a solution, ecological momentary assessments record repeated pain experiences throughout the day in the natural environment.

**Objective:**

The aim of this study was to examine the temporal association between ecological momentary assessments of pain and GPS metrics in older adults with symptomatic knee osteoarthritis by using a smartwatch platform called Real-time Online Assessment and Mobility Monitor.

**Methods:**

Participants (n=19, mean 73.1 years, SD 4.8; female: 13/19, 68%; male: 6/19, 32%) wore a smartwatch for a mean period of 13.16 days (SD 2.94). Participants were prompted in their natural environment about their pain intensity (range 0-10) at random time windows in the morning, afternoon, and evening. GPS coordinates were collected at 15-minute intervals and aggregated each day into excursion, ellipsoid, clustering, and trip frequency features. Pain intensity ratings were averaged across time windows for each day. A random effects model was used to investigate the within and between-person effects.

**Results:**

The daily mean pain intensities reported by participants ranged between 0 and 8 with 40% reporting intensities ≥2. The within-person associations between pain intensity and GPS features were more likely to be statistically significant than those observed between persons. Within-person pain intensity was significantly associated with excursion size, and others (excursion span, total distance, and ellipse major axis) showed a statistical trend (excursion span: *P*=.08; total distance: *P*=.07; ellipse major axis: *P*=.07). Each point increase in the mean pain intensity was associated with a 3.06 km decrease in excursion size, 2.89 km decrease in excursion span, 5.71 km decrease total distance travelled per day, 31.4 km^2^ decrease in ellipse area, 0.47 km decrease ellipse minor axis, and 3.64 km decrease in ellipse major axis. While not statistically significant, the point estimates for number of clusters (*P*=.73), frequency of trips (*P*=.81), and homestay (*P*=.15) were positively associated with pain intensity, and entropy (*P*=.99) was negatively associated with pain intensity.

**Conclusions:**

In this demonstration study, higher intensity knee pain in older adults was associated with lower life-space mobility. Results demonstrate that a custom-designed smartwatch platform is effective at simultaneously collecting rich information about ecological pain and life-space mobility. Such smart tools are expected to be important for remote health interventions that harness the variability in pain symptoms while understanding their impact on life-space mobility.

## Introduction

The world population of adults aged 65 years or older is rapidly growing [1]. This phenomenon, unprecedented in history, highlights a need to maintain and promote programs that manage chronic diseases and symptoms causing increased risk of loss of mobility and disability. The National Center for Health Statistics [2] reports that, of adults 65-75 years old, 30% and 14.3% have physical impairments and difficulty walking one quarter-mile (approximately 400 m), respectively. Rates are higher in those older than 75 years—48.6% have physical impairments, and 27.7% have difficulty walking one quarter-mile (approximately 400 m). These impairments have a significant negative impact on life-space mobility—the daily activities and geographical area in which people engage. As a result, many older adults anchor to their houses [3]. Osteoarthritis is the most common age-related joint disease in the United States, affecting over 30 million US adults [4]. Pain associated with osteoarthritis is accompanied by a reduction in daily functioning, limitations in walking, and increased risk of overt disability. Pain experiences have within- and between-person variability due to physiological, medical, behavioral, and environmental differences [5]. This variability is complex and cannot be captured with traditional recall-based pain surveys. As a solution, ecological momentary assessments (EMA) record repeated pain experiences throughout the day in a person's natural environment. It minimizes retrospective [6,7] and recent-experience bias [8,9]. However, there are drawbacks as the EMA tools that utilize paper surveys or dedicated digital boxes tend to be intrusive, cannot be easily customized, and are not wearable. In prior work [10], microinteraction EMAs—in which people are prompted with questions, similar to those of ROAMM, that can be understood at a glance and answered in a few seconds—were developed on smart watches and compared to less frequent EMA prompts on smartphones; researchers found that although prompts on the watch were 8 times more frequent than those on the phone, participants adhered 35% more to microinteraction EMAs on the watch. Participants also responded to EMAs in less time and reported the EMAs to be less distracting on the watch than those on the phone [11]. Therefore, EMAs on a smartwatch might serve as an excellent approach for enhancing adherence.

Mobility within the perspective of life-space can be described as the habitual movement of individuals [12-14]. Life-space mobility includes spatial size and frequency of interaction with the surrounding environment. The construct is influenced by physical function and spatial extent of movement, but also the cognitive, psychological, social, and environmental disposition of an individual. Life-space mobility has been measured using various methods [12,13,15,16]. Life-Space Diary, introduced by May et al [13] in 1985, was the first measure. It asked participants to report daily their zone out of 5 predefined concentric zones, referenced to their bedrooms. Similarly, Life-Space Questionnaire, introduced by Stalvey et al [14] in 1999, consisted of 9 yes or no questions asking whether a participant was in a certain region within their environment in the last 3 days. Life-Space Assessment, introduced by Baker et al [12] in 2003, added another perspective by documenting how far and how often an individual travels to predefined regions within their environment, while also considering any assistance needed during mobility. However, there remain issues with life-space mobility assessment—paper-based and recall of information are an added burden on participants and introduce more challenges related to adherence and recall bias.

The use of personal devices such as smartphones and smartwatches is growing rapidly in both young and older adult population groups. According to the International Data Corporation Worldwide Quarterly Wearable Device Tracker, smartwatches accounted for 44.2% of the wearable market in 2018; this is expected to rise to 47.1% by 2023 [17]. The widespread use of wearables and their high computational and sensory capabilities provide a platform to reach and interact with a large share of population, particularly individuals with medical conditions. This is highly significant due to the ability to monitor individuals continuously and intervene whenever and wherever medical conditions occur [18]. It also opens new opportunities to link complex states in a temporal manner.

In this demonstration study, we used a custom-designed smartwatch platform called Real-time Online Assessment and Mobility Monitor (ROAMM) that synchronizes EMA of pain experiences with GPS data to examine their temporal associations in older adults with symptomatic knee osteoarthritis. We hypothesized that higher pain experiences would be associated with lower life-space mobility features.

## Methods

### Study Population

This study was approved by the University of Florida institutional review board (UFIRB 201601858), and written informed consent was obtained from all participants. We enrolled 19 older adults. Inclusion criteria were age ≥65 years and diagnosis of unilateral or bilateral symptomatic knee osteoarthritis. Exclusion criteria were failure or inability to provide informed consent; diagnosis of dementia; and being unable to communicate because of severe hearing loss or speech disorder. A convenience sample was drawn from a population of older adults with knee osteoarthritis. Two participants were not interested in participating after being informed about the study. Each participant received compensation of a US $50 gift card. Table 1 shows the descriptive characteristics of participants.

**Table 1:** Participants’ descriptive characteristics.

Characteristics			Value (n=19)
Age (years), mean (SD)			73.1 (4.8)
**Gender, n (%)**			
	Male	6 (32)	
	Female	13 (68)	
BMI (kg/m^2^), mean (SD)			28.23 (4)
**Ethnicity, n (%)**			
	White	15 (79)	
	African American	3 (16)	
	Asian	1 (5)	
**Education, n (%)**			
	Graduate	10 (53)	
	College	6 (32)	
	High school	2 (10)	
	Declined to respond	1 (5)	
**Live alone, n (%)**			
	Yes	4 (21)	
	No	15 (79)	
**Housing, n (%)**			
	Single Family Home	16 (84)	
	Other	1 (5)	
	Other (mobile home, boat)	2 (11)	

### Ecological Momentary Assessment of Pain Using ROAMM

ROAMM was developed at the University of Florida to enable real-time capture of patient-generated information—wearable sensor data collected simultaneously with symptom EMAs. For this study, EMA of pain was evaluated using the 11-point Box Scale (0=no pain, 1-2=mild pain, 3-5=moderate pain, 6=severe pain, 7-9=very severe pain, 10=worst possible pain), a valid and reliable numerical rating scale [19,20]. Participants were instructed about the anchors.

**Figure 1:**
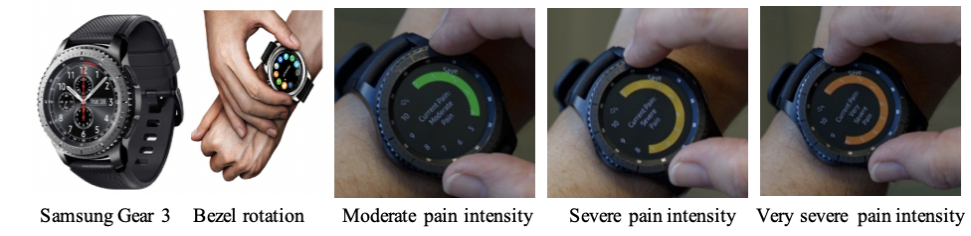
ROAMM app on Samsung Gear 3. Participants rotate the bezel on the watch to select the intensity on a scale from 0 to 10 and the color changes accordingly.

Participants were prompted about their pain intensity at random times in the morning (8 AM to noon), afternoon (noon to 4 PM), and evening (4 PM to 8 PM). The smartwatch application also captures GPS coordinates (latitude and longitude) every 15 minutes throughout the day. Data were transferred every 15 seconds and stored securely in a remote server. The application interface was developed after holding a focus group as explained by Manini and colleagues [21]. ROAMM architecture is explained in detail in our published papers [22,23] (Figure 1). shows ROAMM app for answering a pain prompt on a smartwatch (Samsung Gear 3, Samsung Group).

### GPS Data Collection and Feature Extraction

In this demonstration study, there were some technical difficulties during the initial phase of data collection. These difficulties included weak GPS signal coverages in some places and data transmission problems. The watch required manual checks on mobile networks, roaming, and location services. These issues were discovered and solved during the data collection process. However, data quality checks during the analysis revelated that insufficient GPS data for 9 out of 28 participants. Participants with missing data were similar age (mean 73.3, SD 6.1 years old) and female proportion (7/9, 77.8%) compared to the 19 participants in our paper. Thus, we believe the missing data were randomly lost and did not cause a selection bias.

GPS, the global positioning system, is a navigation utility that furnishes the position of a receiver by measuring its distance from a number of satellites. GPS has been used in the health care domain in behavior [24,25] and gerontological research [26].

**Figure 2:**
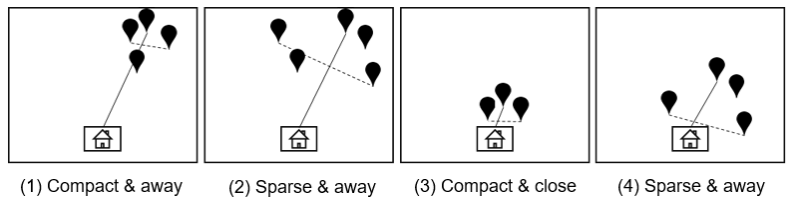
Illustration of possible travel patterns using excursion size and span features. The solid line represents excursion size, and the dashed line represents excursion span.

Excursion features included excursion size, excursion span, and total distance. Excursion size is the farthest distance an individual travels from home within a specific time window. Excursion span is the farthest distance between all locations away from home. These features provide an individual’s travel pattern that can be generally described as (1) compact and away from home; (2) sparse and away from home; (3) compact and close to home; and (4) sparse and close to home (Figure 2). Total distance provides overall view of mobility by summing between all the location points.

**Figure 3:**
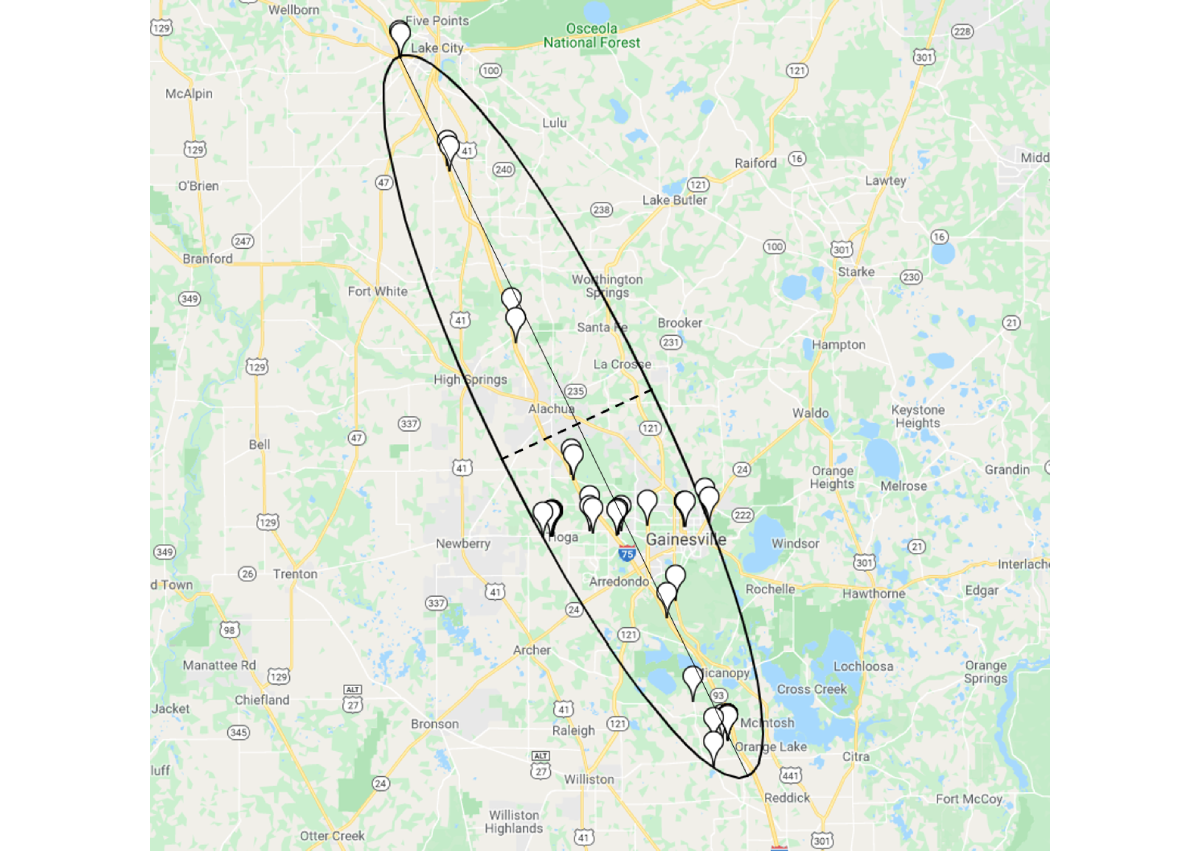
An illustration of the ellipse encompassing all the GPS coordinates for a participant during a 1-day time frame. The dashed line passing through the center represents the ellipse minor axis, and the solid line passing through the center represents the ellipse major axis.

Ellipsoid features used a spanning ellipsoid (or ellipsoid hull), which is defined as the minimum area that encompasses all points in 2 dimensions. We used this method to draw an ellipse such that all GPS coordinates lie inside or on the boundary of the ellipse. We aggregated 3 features from the ellipse: (1) ellipse minor axis, which is the shortest diameter passing through the center of the ellipse; (2) ellipse major axis, which is the longest diameter passing through the center of the ellipse, and (3) area of the ellipse (Figure 3).

Clustering features provided information on where individuals spend most of their time. This is essential to understand the variability in locations. We used a distance-based clustering mechanism, where nearby locations are clustered together. Each cluster has a centroid, and the distance from the centroid determines the membership of a coordinate in that cluster. We used an adaptive *k*-mean algorithm to cluster locations, which does not require a predefined number of clusters that the conventional *k*-mean algorithm requires. Before providing GPS coordinates to the adaptive *k*-mean clustering algorithm, we classified them into stationary and moving coordinates by calculating the time derivative at each location. When the time derivative was <1 km/h), the GPS coordinate was considered stationary. Only stationary points were considered as input to the clustering algorithm. We ran a simulation to find the optimal number of clusters, with a threshold of 500 m from the cluster’s centroid as an inclusion criterion for each cluster. We started with one cluster and gradually increased the number of clusters until all GPS coordinates were assigned to a specific cluster. After clustering all points, we aggregated 2 relevant features: number of clusters and entropy. The number of clusters is simply a count of the generated clusters. The entropy provides information on the distribution of time in different clusters. Entropy measures the degree of disorder or the level of uncertainty in the information theory. In our analysis, entropy was used to measure the level of uncertainty in the time spent in different clusters. Entropy is calculated using the following formula:


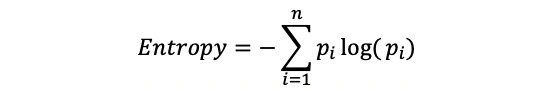


where *p*_i_ is the percentage of time a participant spends at cluster *i*, and *p*_i_ is between (0,1].

**Figure 4:**
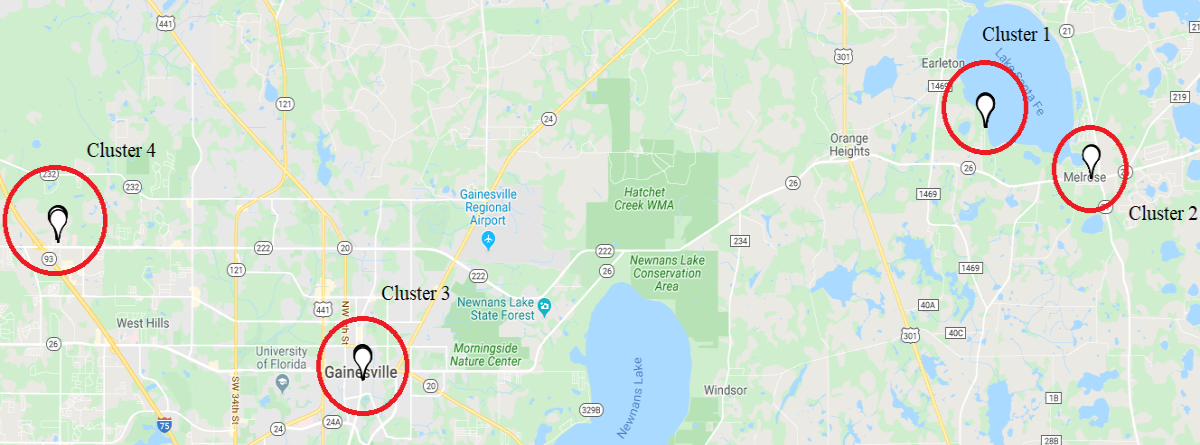
Illustration of clustering for a participant’s coordinates within a 1-day time frame. A total of 4 clusters are shown on the map, where each cluster contains a collection of GPS coordinates.

A low entropy value means a lower level of uncertainty and that the participant spent most of the time at one location, which is an indication of lower life-space mobility (Figure 4).

Frequency of trips and homestay percentage provided information about the number of trips away from home and time spent at home, respectively. First, we classified the GPS coordinates as home or away from home, then calculated the 2 features accordingly. Homestay is represented as a fraction between 0 and 1. It is the ratio of the number of GPS coordinates within the home radius (ie, 100 m) to the total number of coordinates. Homestay is considered 0 (or 0% when all the GPS coordinates are outside home in a given time period of interest, and 1 (or 100%) when all the GPS coordinates are within the home radius. A trip is calculated when a sequence of GPS coordinates—home, away from home, home—occurs chronologically. The number of trips occurring within a specific time window are summed to yield the frequency of trips.

### Statistical Analysis

We evaluated the relationship between EMA of pain (predictor) and the measures of life-space mobility using GPS features (outcomes). Pain intensity ratings were averaged across day-windows. This was done to better connect to the day-based frequency of measurement for the GPS features. In addition to that, we graphically expressed pain intensities into 2 groups: low pain (<2) and high pain (≥2), but statistical comparison was not performed.

A 2-level random effects model (participant and day) was used to account for repeated measurements. The model was fit after disaggregating the within and between-persons effects. Parceling these effects allows a more in-depth understanding about the association between GPS features and EMA pain. The approach used person-mean centering around the grand mean (between-person effect) and the within person effect (each person-specific mean for the time varying covariate) [27-29]. We used the xtcenter command (Stata/MP; version 16.0 for Windows; StataCorp LLC) and entered terms for within-person and between-person effects into the model. The model was also adjusted for age, living alone, and gender covariates as fixed effects. An independent-covariance structure, which was confirmed as the most efficient without loss of model fit using the Akaike information criterion, was used in all models. All analyses were conducted using Stata/MP. Statistical significance was confirmed at the *P*≤.05 level. Because this study is a demonstration project, *P*≤.10 was considered as a trend effect.

## Results

**Figure 5:**
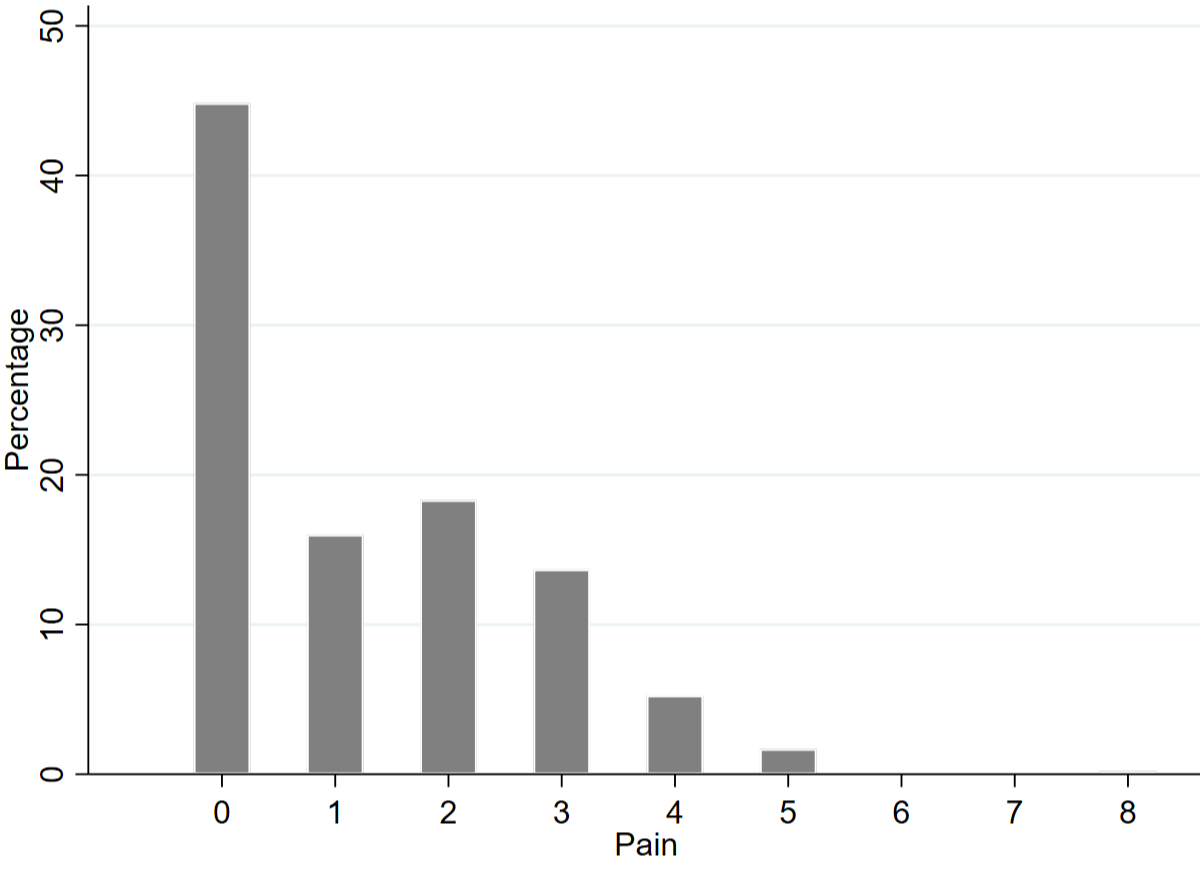
Histogram showing pain distribution.

Participants wore the smartwatch for a mean of 13.16 (SD 2.94) days and responded to a mean of 82% of pain prompts. Figure 5 shows the distribution of reported pain intensities by all participants. A pain intensity rating of 0 was the most common intensity and the highest was 8. The mean pain intensity for the low pain group was 0.26 (SD 0.44) and for the high pain group was 2.78 (SD 0.93). Descriptive characteristics of the life-space mobility features are listed in Table 2. Multimedia Appendix 1 shows that some GPS features were intercorrelated—there were strong correlations between excursion features and ellipsoid features and weak correlations between the remaining features.

**Table 2:** Descriptive characteristics of life-space mobility features.

Features	Mean	SD	Median	Kurtosis
Excursion size (km)	11.35	18.97	18.99	17.87
Excursion span (km)	11.33	19.73	4.72	15.34
Total distance (km)	23.14	38.21	8.67	10.43
Ellipse area (km^2^)	104.89	342.11	8.00	42.39
Ellipse minor axis (km)	3.41	4.65	1.49	5.87
Ellipse major axis (km)	14.59	23.42	7.34	16.75
Frequency of trips	2.88	4.20	2	8.46
Homestay percentage	0.66	0.31	0.74	–0.31
Number of clusters	2.02	1.08	2	0.86
Entropy	0.30	0.34	0.20	0.05

**Figure 6:**
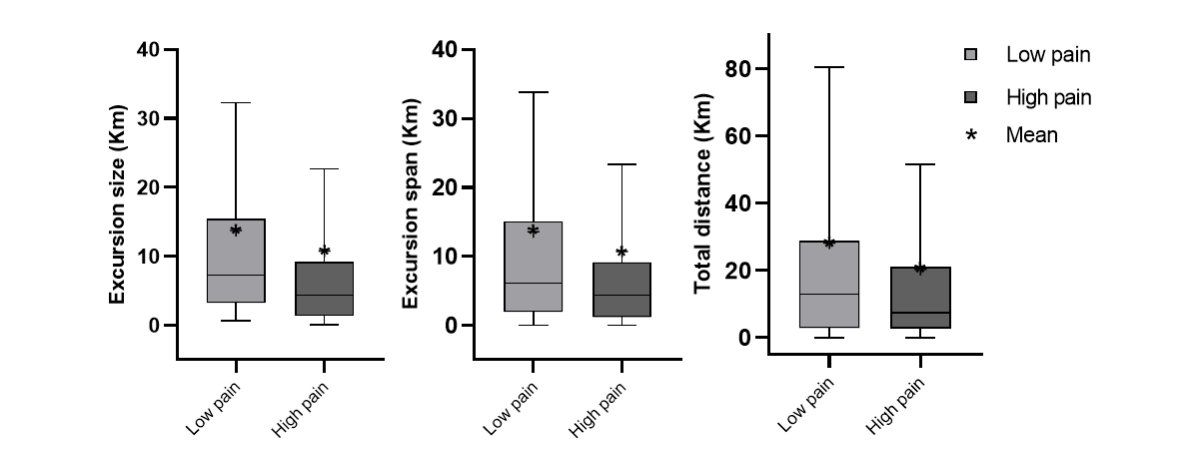
Distance features including excursion size, excursion span, and total distance for each pain group.

**Figure 7:**
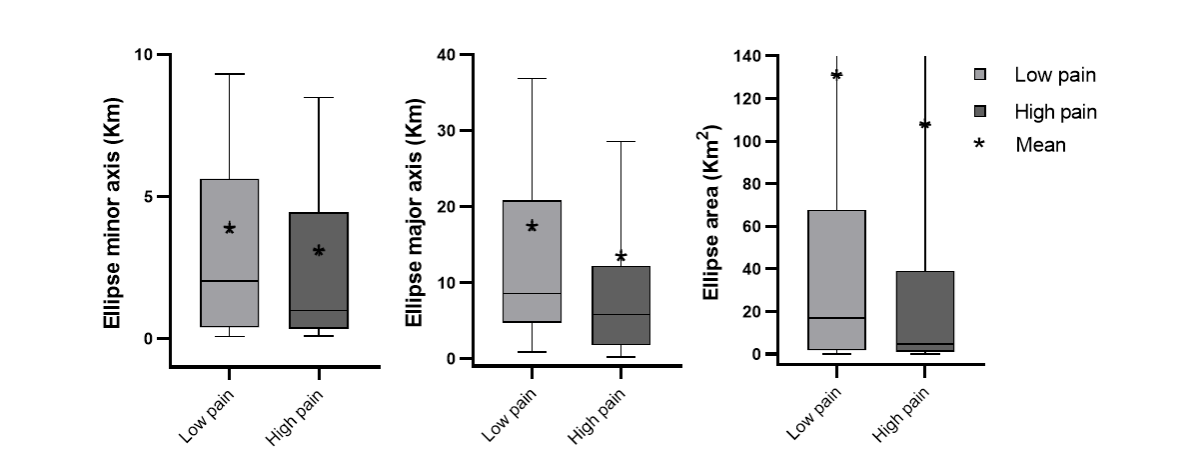
Ellipse minor axis, ellipse major axis, and ellipse area for each pain group.

**Figure 8:**
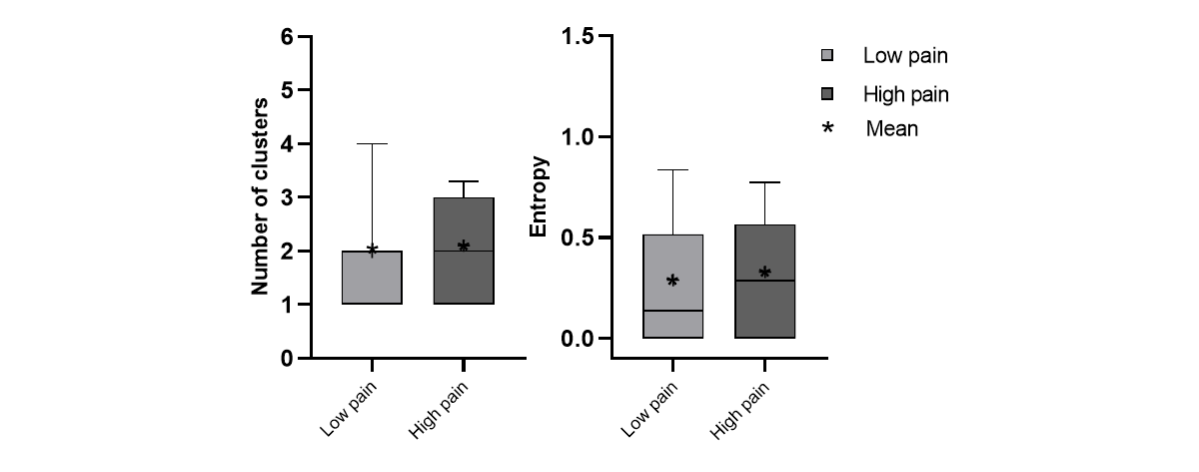
Clustering features including number of clusters and entropy for each pain group.

**Figure 9:**
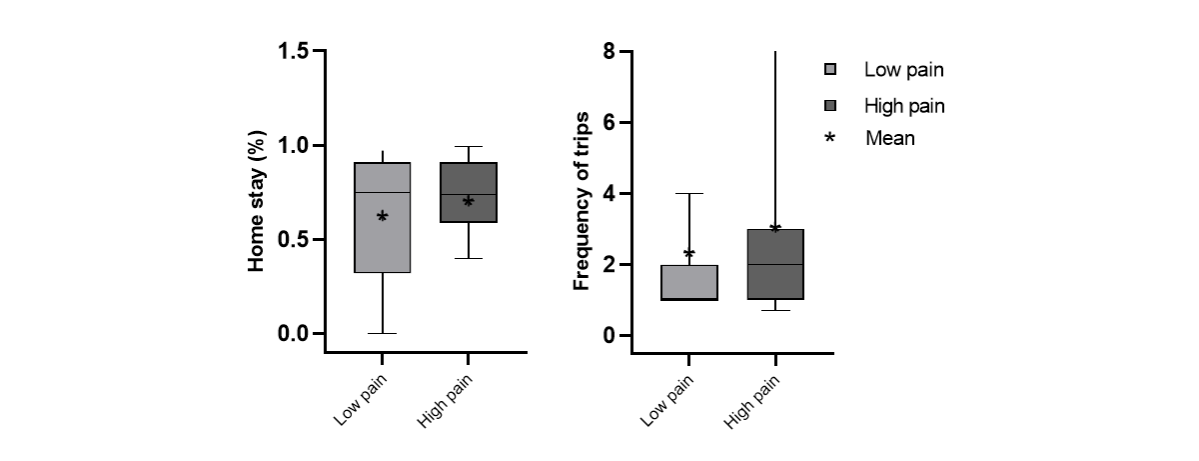
Frequency of trips and homestay percentages for each pain group.

The median and mean of the daily GPS features of the low pain group were generally higher than those of the high pain group for excursion features (Figure 6) and ellipsoid features (Figure 7). The results of the mixed-effect model are shown in Table 3. There were no between-person effects of pain intensity on GPS features (Figure 8 and Figure 9), but within-persons association were predominant. The majority of GPS features (7 out of 10) indicated that having high pain was associated with a lower value (ie, life-space mobility). Among these GPS features, within-person pain intensity was significantly associated with excursion size, and others (excursion span, total distance, and ellipse major axis) showed a statistical trend (*P*<.10).

**Table 3:** Mixed effect association between pain and GPS features adjusted for age, living alone, and gender covariates.

GPS features and mixed-model effects^a^		Coefficient	SE	*P*>|*z*|	95% CI
**Excursion size**					
	Between	–3.79	4.52	.40	–12.65, 5.06
	Within	–3.06	1.58	.05	–6.16, 0.04
**Excursion span**					
	Between	–3.18	4.71	.50	–12.41, 6.05
	Within	–2.89	1.65	.08	–6.11, 0.35
**Total distance**					
	Between	–11.92	9.16	.19	–29.94, 5.95
	Within	–5.71	3.17	.07	–11.93, 0.51
**Ellipse area**					
	Between	–58.23	86.66	.50	–228.07, 111.62
	Within	–31.42	27.67	.26	–85.65, 22.81
**Ellipse minor axis**					
	Between	–1.31	1.08	.23	–3.43, 0.82
	Within	–0.47	0.38	.22	–1.21, 0.28
**Ellipse major axis**					
	Between	–4.86	5.57	.38	–15.78, 6.06
	Within	–3.64	1.97	.07	–7.50, 0.22
**Frequency of trips**					
	Between	0.51	0.50	.31	–0.47, 1.49
	Within	0.05	0.22	.81	–0.38, 0.49
**Homestay percentage**					
	Between	–0.003	0.04	.94	–0.08, 0.08
	Within	0.03	0.02	.15	–0.01, 0.06
**Number of clusters**					
	Between	0.11	0.24	.63	–0.35, 0.58
	Within	0.03	0.08	.73	–0.14, 0.19
**Entropy**					
	Between	–0.04	0.07	.53	–0.18, 0.10
	Within	–0.0003	0.03	.99	–0.05, 0.05

^a^Values represent the within- and between-person effect.

## Discussion

This study used a customized smartwatch app for EMA of pain and life-space mobility as a demonstration project. Previously, these constructs have not been coupled into a single platform that permits synchronizing of symptoms with objective measures of mobility in the natural environment. The results suggest that EMA of pain is negatively associated with most but not all life-space mobility features. Importantly, within-person effects, but not between-person effects, were more likely to be statistically significant. In general, older adults with confirmed knee osteoarthritis had lower life-space mobility, when pain intensity exceeded 2 out of 10. The results confirm the feasibility and analytic procedures for using smartwatch technology to harnesses sensor data alongside EMA of clinically relevant symptoms.

Chronic pain, such as pain from symptomatic knee osteoarthritis, is dynamic [30]. The variability of pain within and between days makes it hard to fully capture pain experience [5]. There has been long-standing interest in understanding daily pain [5,31]. Earlier endeavors relied on patient recall of pain, which is susceptible to recall bias and lack of ecological validity of the assessment [32]. EMA is an alternative tool to allow researchers to capture and assess a person's pain multiple times in the person's natural environments. Electronic handheld devices have provided additional features to EMA research via their ability to capture moment-by-moment data generated by their built-in sensors, allowing an in-depth understanding of the impact that pain experiences have on mobility patterns (eg, life-space mobility). Studies [33-36] have used handheld devices (eg, smartphones and iPod) for EMA of pain; however, the use of these devices was limited to electronically record participants’ diaries without utilizing the built-in sensors, and no studies have used smartwatches for data collection.

In our study, 10 semantically meaningful features were extracted from the GPS coordinates according to previous work [24-26] and redefined as life-space mobility metrics. We chose to separate within- and between-person effects to study the associations of pain intensity ratings on GPS features. This was done because typical coefficients from random effects models represent a blend of both [37]. The within-person associations demonstrate that pain and life-space mobility relate to each other on an individual level. This is important because previous research demonstrates that life-space mobility is lower in people reporting higher levels of pain [37]. The within-person associations found in this study not only support the previous between-person findings, they also support the notion that pain-related interventions are likely to have an impact on an individuals’ life-space mobility. Specifically, excursion features were negatively associated with pain intensity. Among these features, within-person pain intensity was significantly associated with excursion size, and showed a statistical trend (*P*<.10) with excursion span and total distance. Each point increase in the mean pain intensity was associated with a 3.06 km decrease in excursion size, 2.89 km decrease in excursion span, and 5.71 km decrease in total distance. This suggests overall travel patterns are closer to home and more compact when older adults are experiencing a higher mean pain intensity.

The spanning ellipsoid, which summarizes the GPS coordinators into 2 dimensions, was negatively associated with within-pain intensity. The ellipsoid features represent a close approximation of the life-space concept (ie, reaching circular levels away from home). Ellipse major axis, which indicates the maximum distance across life-space, was significantly associated with pain intensity. Each point increase in the mean pain intensity was associated with 31.4 km^2^ decrease in ellipse area, 0.47 km decrease in ellipse minor axis, and 3.64 km decrease ellipse major axis. Notably, the ellipse tends to have a smaller area, length, and width with higher pain intensity, which is similar to our observation about excursion features. Point estimates suggest that higher intensities of pain may constrain individuals to their home and limit the number of places they can visit.

Location clustering provides information about the distribution of places individuals spend outside of their homes. The number of clusters and entropy both contribute to understanding the variability of places visited by participants. While not statistically significant, the directionality of the coefficients indicated that higher pain intensities were associated with a higher number of places an individual stays at (stationary places). In other words, higher pain appears to be associated with spending more time at a lower number of locations, but this needs to be confirmed in larger samples.

The directionality of the point estimates demonstrated that the frequency of trips was higher when pain intensity was high. Although this may seem counterintuitive, coupled with the other results, it appears that these frequent trips were close to home. Similarly, point estimates for homestay percentage were positively associated with pain intensity. Given the weak associations of these features, trip frequency and homestay percentage may not be useful features for understanding the impact of pain on life-space mobility.

The association between pain and life-space mobility has not been widely studied, and more research is needed in this regard [38,39]. Despite the lack of relevant research, our results agree with those of Rantakokko et al [38] and Liddle et al [40], where life-space mobility was found to be negatively associated with pain. Rantakokko et al [38] examined the association between life-space mobility and multiple outcomes, including pain, in patients with Parkinson disease. They followed a paper-based questionnaire and assessed life-space mobility using life-space assessment. They found that life-space mobility is negatively associated with pain [38]. Similarly, Liddle et al [40] examined life-space mobility among patients with Parkinson disease using GPS on smartphones. They found that people with more symptoms spend more time at home and travel shorter distances.

Other studies have found strong associations between life-space mobility and depression [25], visual impairment [41], and personal and social characteristics [42,43] using GPS. Among these studies, only Cornwell et al [42] used smartphones for GPS tracking and EMA collection to examine the social environments relevant to older adults’ everyday lives, where they found that certain activities such as exercising, shopping, socializing, and social activities were likely to take place outside of residential tracts. These studies [25,41-43] show the important role GPS features play, when coupled with other outcomes, in understanding individuals’ behavior and their experience in natural environment and the importance of wearables in linking complex states in a temporal manner.

This demonstration study provided insights on the potential relationship between life-space mobility and pain in older adults with symptomatic knee osteoarthritis by utilizing smartwatches. Our results demonstrate that a custom-designed smartwatch platform was effective at simultaneously collecting rich information about ecological pain and life-space mobility. ROAMM could potentially help clinicians in assessing pain or other patient-reported outcomes in patients’ natural environments, while continuously collecting relevant sensory data. Though our results point to interesting insights in understanding the relationship between life-space mobility and EMA of pain, our study had limitations. The sample size was not large enough to generalize and infer causality between life-space mobility and pain. Additionally, the overall pain reported by participants was low, with the majority reporting a pain intensity less than 4. In the future, we aim to recruit a larger sample size with more diversity in terms of pain intensity.

The major goal of this study was to demonstrate that a smartwatch platform—ROAMM—could be used to collect EMA of pain with concurrent mobility tracking via GPS for life-space mobility assessment in older adults with symptomatic knee osteoarthritis. Point estimates from other life-space mobility features confirm that the directionality of associations is plausible and provides initial evidence for their utility in future studies. In general, it appears that higher intensities of pain intensity tend to limit their life-space mobility by either constraining them to their residence or limiting their excursion lengths. This area of research is still in its infancy, but with apps similar to ROAMM, the demand for these tools is expected to increase remote health endeavors that are gaining significant momentum in health care. Such connected technologies have a potentially important role giving practitioners information about their patients' behaviors, symptoms, and health condition sequalae in their patients' natural environments.

## Appendix

Multimedia Appendix 1 Feature correlation matrix.

## Abbreviations

EMA: ecological momentary assessment
GPS: global positioning system
ROAMM: Real-time Online Assessment and Mobility Monitor
