# Supplementary material for: The Temporal Relationship Between Ecological Pain and Life-Space Mobility in Older Adults With Knee Osteoarthritis: A Smartwatch-Based Demonstration Study
Source: JMIR Mhealth Uhealth. 2021 Jan 13;9(1):e19609. doi: 10.2196/19609 (PMC7840291; doi:10.2196/19609)
Supplement: Multimedia Appendix 1 [file mhealth_v9i1e19609_app1.docx]

### Multimedia Appendix 1

Correlation matrix of the GPS features.


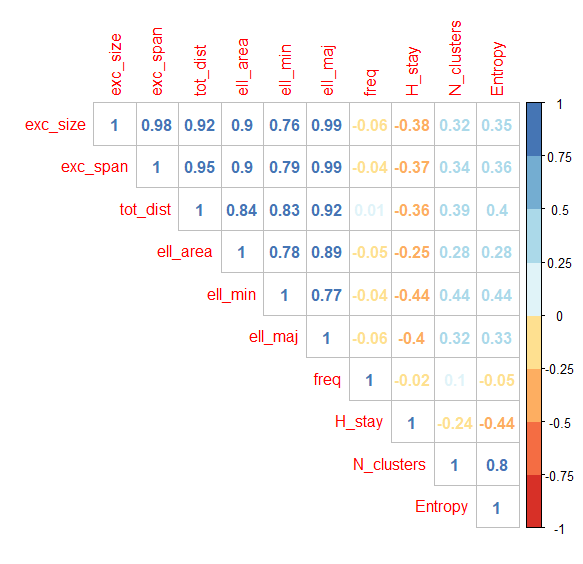


Correlation matrix of the GPS features. exc_size: excursion size; exc_span: excursion span; tot_dist: total distance; ell_area: ellipse area; ell_min: ellipse minor axis; ell_maj: ellipse major axis; freq: frequency of trips; H_stay: homestay percentage; N_clusters: number of clusters; Entropy: entropy.
